# Intravenous Infusion of Dexmedetomidine Combined Isoflurane Inhalation Reduces Oxidative Stress and Potentiates Hypoxia Pulmonary Vasoconstriction during One-Lung Ventilation in Patients

**DOI:** 10.1155/2015/238041

**Published:** 2015-07-26

**Authors:** Rui Xia, Jinjin Xu, Hong Yin, Huozhi Wu, Zhengyuan Xia, Daiwei Zhou, Zhong-yuan Xia, Liangqing Zhang, Haobo Li, Xiaoshan Xiao

**Affiliations:** ^1^Department of Anesthesiology, First Affiliated Hospital, Yangtze University, Jingzhou 434000, China; ^2^Department of Anesthesiology, Wuhan University Renmin Hospital, Wuhan 430060, China; ^3^Department of Cardiothoracic Surgery, Fifth Affiliated Hospital of Zunyi Medical College, Zhuhai 519100, China; ^4^Department of Anesthesiology, The Second Affiliated Hospital & Yuying Children's Hospital of Wenzhou Medical University, Wenzhou, Zhejiang 325000, China; ^5^Department of Anesthesiology, Affiliated Hospital of Guangdong Medical College, Zhanjiang, Guangdong 524001, China; ^6^Department of Anesthesiology, The University of Hong Kong, Hong Kong; ^7^Department of Anesthesiology, Guangdong No. 2 Provincial People's Hospital, Guangdong Provincial Emergency Hospital, Guangzhou, Guangdong 510317, China

## Abstract

Inhalation anesthetic isoflurane inhibits hypoxia pulmonary vasoconstriction (HPV), while dexmedetomidine (Dex) could reduce the dose of isoflurane inhalation and potentiate HPV, but the mechanism is unclear. Inhibition of reactive oxygen species (ROS) production can favor HPV during one-lung ventilation (OLV). Similarly, nitric oxide (NO), an important endothelium-derived vasodilator in lung circulation, can decrease the regional pulmonary vascular resistance of ventilated lung and reduce intrapulmonary shunting. We hypothesized that Dex may augment HPV and improve oxygenation during OLV through inhibiting oxidative stress and increasing NO release. Patients undergoing OLV during elective thoracic surgery were randomly allocated to either isoflurane + saline (NISO, *n* = 24) or isoflurane + dexmedetomidine (DISO, *n* = 25) group. Anesthesia was maintained with intravenous remifentanil and inhalational isoflurane (1.0–2.0%), with concomitant infusion of dexmedetomidine 0.7 *μ*gkg^−1^h^−1^ in DISO and saline 0.25 mL kg^−1^h^−1^ in NISO group. Hemodynamic variables or depth of anesthesia did not significantly differ between groups. Administration of Dex significantly reduced Qs/Qt and increased PaO_2_ after OLV, accompanied with reduced lipid peroxidation product malondialdehyde and higher levels of SOD activity as well as serum NO (all *P* < 0.05 DISO versus NISO). In conclusion, reducing oxidative stress and increasing NO release during OLV may represent a mechanism whereby Dex potentiates HPV.

## 1. Introduction

With the popularity of video-assisted thoracic surgery, the requirement for one-lung ventilation (OLV) has been increasing. OLV is used to provide a good surgical field and protect normal lungs from hemorrhage or abscess caused by affected lung [1]. However, OLV can induce ventilation-perfusion mismatch and pulmonary arteriovenous shunt in the nonventilated lung that can cause hypoxemia [2]. Hypoxic pulmonary vasoconstriction (HPV) is an important protective mechanism by which blood flow is diverted from the nonventilated lung toward a better ventilated region, thereby maintaining adequate arterial oxygenation [3]. Inhalational anesthetic sevoflurane and isoflurane have been shown to inhibit HPV and thereby increase hypoxemia [4]. Dextral dexmedetomidine (Dex) is a new highly selective alpha2-adrenergic receptor agonist which has been increasingly used both in the intensive care unit and also perioperatively as an adjunct to general anesthesia. Dex has been shown to reduce the dose of the inhalational and intravenous anesthetics [5, 6] and to reduce anti-inflammatory properties against sepsis induced lung injury [7] and in bleeding-induced multiple organ dysfunction syndrome in rats [8]. Our recent study showed that intravenous infusion of Dex combined with inhalation of isoflurane potentiated HPV and thereby improved oxygenation during OLV [9]. However, the underlying mechanism remains to be elucidated.

Dex can induce vasoconstriction by activating alpha2-adrenoreceptor at a dose dependent manner [10]. It was reported that Dex even at a concentration 5 to 15 times lower than the clinically recommended plasma target concentration (0.4 to 1.2 ng/mL) could induce vasoconstriction [11]. Also, Dex at the clinical concentration can decrease the redistribution of pulmonary blood flow from the ventilated to the nonventilated lung [9]. How did Dex maintain or augment the small pulmonary arteries contraction in the nonventilated lung and/or induce vasodilation in the ventilated lung? The ventilated lung should have the same alpha2- adrenoreceptors as the nonventilated lung in a specific person. This suggests that Dex may have conferred its effects through mechanisms other than a direct vasoconstriction modulator by activation of alpha2-adrenoceptor agonist.

Current evidence suggests that reactive oxygen species (ROS) and oxidative stress play a fundamental role in the regulation of HPV [12, 13]. Hypoxia is monitored by the pulmonary arteries smooth muscle cells (PASMCs) [14, 15]: during hypoxia, inhibition of ROS production and changes in the ratios of cytosolic reducing cofactors (GSH/GSSG, NADH/NAD^+^, and NADPH/NADP^+^) within human PASMCs activate voltage-gated K^+^ channels in PASMCs, resulting in membrane depolarization and opening of L-type Ca^2+^ channels, which increases intracellular Ca^2+^ concentration and then elicits compensatory pulmonary artery constriction in hypoxic lung. Although inhibition of ROS production favors HPV during acute hypoxia, clinical studies found that during one-lung ventilation systematic lipid peroxidation product malondialdehyde (MDA) level was significantly increased which indicates increased oxidative stress [16]. During one-lung ventilation, ROS can be produced from multiple sources including mechanical ventilation, surgical trauma, manipulated lung tissue, and hyperoxia in ventilated lung [17]. Hypoxia also damps the levels of endogenous antioxidant enzyme superoxide dismutase (SOD) [18, 19], which plays an important role in balancing ROS generation and the overall tissue antioxidant capacity. Therefore, decreased SOD activity aggravates oxidative stress, which alleviates HPV effect. Studies found that Dex can decrease oxidative stress during pneumoperitoneum and strengthen the antioxidant defense system [20]. Whether Dex can inhibit oxidative stress during one-lung ventilation and whereby potentiates HPV effect is unclear.

Nitric oxide (NO) is an important endothelium-derived relaxing factor in lung circulation [21], which is produced mainly from human pulmonary endothelial cells (PAECs) in the pulmonary circulation by endothelial nitric oxide synthase (eNOS). Studies found that inhalation of nitric oxide can decrease the regional pulmonary vascular resistance of ventilated lung area, decrease intrapulmonary shunting, and improve arterial oxygenation [22, 23]. However, Takemoto et al. reported that chronic hypoxia is associated with a decrease in eNOS mRNA and protein expression in human PAECs [24]. Alpha2-adrenergic receptor agonist can activate NO production [25]. However, it is unknown whether Dex can activate NO production and decrease intrapulmonary shunting during one-lung ventilation in patients.

Therefore, the current study was designed to test the hypothesis that Dex can inhibit oxidative stress and increase NO production, whereby decreasing intrapulmonary shunting and improving arterial oxygenation in patients under one-lung ventilation.

## 2. Methods

### 2.1. General Information

After approval of the institutional ethical committee and written informed consent, 60 male patients (40–60 years old, ASA I~II, 50~73 kg, 151~175 cm high) undergoing elective thoracic surgery were included in the study. The clinical trial registration number is Chic TROIR-15005784. Exclusion criteria were renal insufficiency, liver dysfunction or ischemic or valvular heart disease, long-term alcohol, opioid, or sedative hypnotic drug addiction and dependency history, and neuropsychiatric diseases.

### 2.2. Group Division

All patients were randomly divided after induction of general anaesthesia into two groups, intravenous infusion of the Dex combined with isoflurane inhalation (DISO group) and the intravenous infusion of normal saline with isoflurane inhalation (ISO group), 30 patients in each group. Both the patients and the anaesthesiologists were blinded to the identity of the study drug (Dex or saline placebo). Study drug (Dex) or placebo was prepared in a 50 mL syringe without identification marked by specialized staff, who does not take part in the process of anaesthesia and the research.

### 2.3. Anaesthesia

Routine monitoring was established on all patients including ECG, noninvasive blood pressure (NIBP), and SPO_2_. Central vena catheterization, radial artery cannulation and module, and electrodes monitor for bispectral index (BIS, Aspect Medical Systems) were also performed in all patients. Anaesthesia was induced with intravenous midazolam 2 mg, fentanyl 4 ug/kg, propofol 2 mg/kg, and vecuronium 0.1 mg/kg. A left-side double-lumen tube was inserted and correct position was assured by auscultation and by fiberoptic bronchoscopy before and after the patient was in the lateral decubitus position. Intermittent positive pressure ventilation (IPPV), mechanical ventilation, was used during one-lung ventilation with tide volume (TV) 8 mL/kg, respiratory rate (RR) 12 bpm, I : E = 1 : 2, FiO2 100%. After bronchial intubation was positioned, patients in DISO group received intravenous infusion of Dex (D-dexmedetomidine liquid, Jiangsu Hengrui Medicine Co., Ltd., production, 100 ug/mL, diluted with normal saline to 50 mL) at 1.0 ug·kg^−1^·h^−1^ over 10 minutes, which was then reduced to 0.7 ug·kg^−1^·h^−1^ and maintained throughout the study; patients in NISO group were given intravenous infusion of 0.25 mL·kg^−1^·h^−1^ saline, and it was reduced to 0.18 mL·kg^−1^·h^−1^ 10 minutes later. During maintenance of anesthesia, all patients were given isoflurane 1.0 to 2.0%, intravenous infusion of remifentanil 0.1~0.2 ug·kg^−1^·min^−1^, and rocuronium to maintain the BIS between 40 to 60. Supplemental vasoactive drugs were used to maintain hemodynamic stability, and the doses of vasoactive drugs (atropine, ephedrine, and urapidil) used in two groups were recorded.

### 2.4. Monitoring

Pulmonary function tests and arterial blood gas analysis for all patients were performed before operation. Arterial blood gases were drawn; central venous blood gas, heart rate (HR), mean artery blood pressure (MAP), BIS values, and intrapulmonary shunt according to the formula Qs/Qt = [(Cc′O2 a CaO2)/(Cc′O2 a CvO2)] × 100% [26] were recorded at 15 minutes during two-lung ventilation (TLV) (TLV-15) and after 10, 20, 30, and 40 min of OLV (OLV-10, OLV-20, OLV-30, and OLV-40). Drager PM8030 was used to measure the concentrations of isoflurane in the inhalation gas (FiIso) and in the exhaled gas (EEIso).

### 2.5. Plasma SOD Activity and MDA Level

Blood sample was collected at TLV-15 and OLV-10, OLV-20, OLV-30, and OLV-40 minutes, respectively, and then plasma was separated by centrifugation. Plasma SOD activity and MDA level were measured by using specific reagents according to the protocols provided by the manufacturer (Nanjing, Jiancheng Bioengineering Institute, China) as described [27, 28], in which the xanthine oxidase method was used for the detection of SOD activity while the thiobarbituric acid was used as substrate for the detection of MDA [29].

### 2.6. Serum NO Concentration

Blood sample was collected at TLV-15 and OLV-30 minutes, respectively, and then serum was separated by centrifugation. Total NO concentration was determined using an indirect method based on measurement of nitrite concentration in serum according to Griess's method [30].

### 2.7. Statistical Analysis

The data were expressed as mean ± standard deviation ($\overset{-}{x}\pm s$). SPSS 13.0 statistical software was used for analysis. The data that meet the analysis of variance between groups comparison were analyzed by ANOVA; pairwise comparison between groups was analyzed by SNK test; one-lung ventilation blood gas analysis of data over time was analyzed by repeated measures analysis of variance. A *P* value less than 0.05 was considered statistically significantly different.

## 3. Results

60 patients were enrolled in the study. 11 patients were excluded from analysis (six in NISO and five in DISO group): in group NISO, 3 patients had BIS value over the range while another 3 cases had SpO2 less than 90% during one-lung ventilation; in group DISO, 2 cases had BIS value over the range and 3 cases had SpO2 less than 90%. Therefore, data from 49 patients were statistically analyzed in this study: 25 in group DISO and 24 in group NISO.

Patients' characteristics are presented in Table 1. There were no differences among groups regarding age, weight, height, ASA physical status, preoperative blood gas, and preoperative pulmonary function.

As shown in Table 2, the values for pH, Hb, PaCO_2_, SaO_2_, and ScvO_2_ did not differ significantly between groups. Initiation of OLV caused a significant decrease in PaO_2_ during conversion from TLV to OLV in both groups and PaO_2_ reached its lowest value at OLV-30 min. The decrease in PaO_2_ in group DISO was less severe as compared to NISO during OLV (*P* < 0.05). However, there was no hypoxemia (too low PaO_2_) recorded in both groups. On changing from TLV to OLV, Qs/Qt% increased significantly in both groups and peaked at OLV-30 min, but the increase of Qs/Qt% in group DISO was less severe as compared with group NISO (*P* < 0.05, Table 2). Heart rate was significantly slower in group DISO than that in group NISO (*P* < 0.05). However, MAP and the use of vasoactive drugs were not significantly different between the two groups (*P* > 0.05) (Table 2).

**Figure 1:**
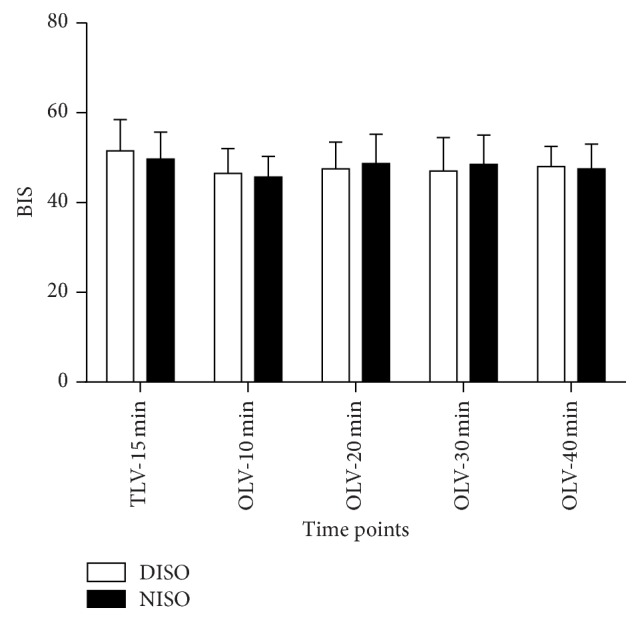
Perioperative time-course alterations of the bispectral index (BIS) in DISO group and NISO group. The values were measured as follows: 15 min after two-lung ventilation (TLV-15), 10 min after one-lung ventilation (OLV-10 min), 20 min after one-lung ventilation (OLV-20 min), 30 min after one-lung ventilation (OLV-30 min), and 40 min after one-lung ventilation (OLV-40 min). Data are presented as median (interquartile range).

**Figure 2:**
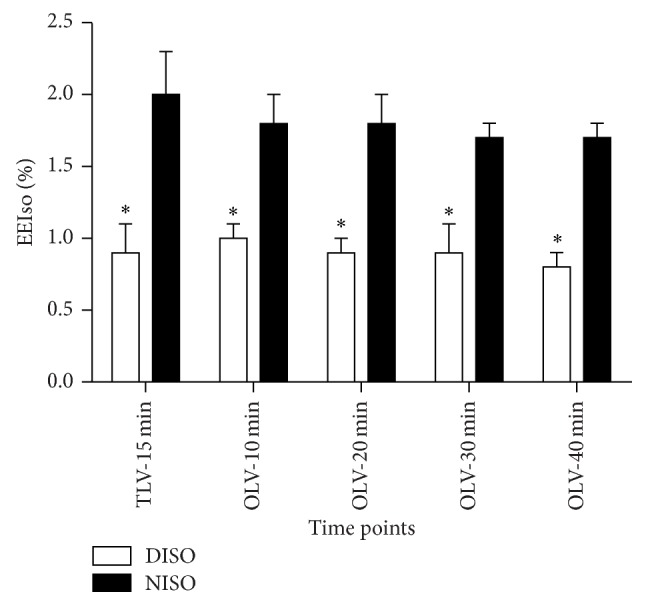
Perioperative time-course alterations of the end-expiratory isoflurane concentration (EEIso) in DISO group and NISO group. The values were measured as follows: 15 min after two-lung ventilation (TLV-15), 10 min after one-lung ventilation (OLV-10 min), 20 min after one-lung ventilation (OLV-20 min), 30 min after one-lung ventilation (OLV-30 min), and 40 min after one-lung ventilation (OLV-40 min). Data are presented as median (interquartile range). ^*^ *P* < 0.05 intergroup comparison between group DISO and group NISO.

BIS values were similar throughout the studied period in each group and between the two groups (Figure 1). FETIso was significantly lower in group DISO throughout the study as compared with group NISO (*P* < 0.05, Figure 2).

The plasma MDA level was about 10% higher in group NISO than in group DISO at TLV-15 min but it was not statistically different (*P* > 0.05, Table 3). On changing from TLV to OLV, the MDA level significantly increased further in group NISO at OLV-30 min (*P* < 0.05 versus TLV-15 and versus DISO, Table 3), while MDA level did not significantly increase in group DISO. Concomitant with the change of MDA level, plasma SOD activity was significantly decreased in group NISO at OLV-30 min, which was remarkably lower than that in group DISO (*P* < 0.05, Table 3), while there was no significant change in SOD activity in group DISO after OLV.

We have observed the changes of Qs/Qt% and PaO_2_ at 5 different time points from TLV-15 min to OLV-40 min and found that, on changing from TLV to OLV, Qs/Qt% increased significantly in both groups and peaked at OLV-30 min while PaO_2_ reached its lowest value at OLV-30 min in both groups. These results suggested that the time point of OLV-30 min may be the key moment for NO releasing, so we detected serum NO at the time point of OLV-30. There was no significant difference in serum NO concentration between two groups at TLV-15 min. After conversion from TLV to OLV-30 min, the serum NO concentration was significantly increased in group DISO (*P* < 0.05, Table 3), but it did not change significantly in group NISO (*P* > 0.05, Table 3), and the values of NO content at OLV-30 min was significantly higher in DISO than in NISO group.

## 4. Discussion

The use of intravenous infusion Dex combined inhalation isoflurane in comparison with isoflurane alone can attenuate the increase in shunt fraction and improve PaO2 in patients undergoing OLV, but the mechanism is unclear. In the current study, we further revealed that plasma MDA level remarkably increased and SOD activity decreased in isoflurane group accompanied with decreased NO concentration, while Dex-isoflurane combination significantly decreased MAD level, maintained SOD activity, and increased serum NO content during OLV. The results support our hypothesis that Dex at clinical dose combined with isoflurane can inhibit oxidative stress and increase NO release compared with volatile anesthetics alone, whereby reducing shunt fraction and improving oxygenation during one-lung ventilation.

The effect of DEX on HPV during OLV is likely dose dependent. Kernan et al. recently reported that Dex administered at a loading dose of 0.3 *μ*g/kg and an infusion rate of 0.3 *μ*g/kg/h did not affect HPV and oxygenation, although the PaO2/FiO2 ratio in patients receiving Dex was relatively higher [31]. However, in our previous [9] and current study, Dex at a higher loading dose of 1 *μ*g/kg and an infusion of 0.7 *μ*g/kg/h, combined isoflurane, significantly limited the increase in pulmonary shunt and the decrease in PaO2 in one-lung ventilated patients, compared with isoflurane alone group. In the current study, DEX-isoflurane treatment decreased plasma MDA content and kept SOD activity, an indication of attenuation of oxidative stress and improvement in the endogenous antioxidative capacity. Furthermore, Dex-isoflurane treatment increase serum NO content, an important endothelium-derived vasodilation factor. These results suggested that DEX should be provided at a dose sufficient to prevent oxidative stress.

ROS are important messengers produced in response to changes in oxygen tension and contribute to the regulation of acute HPV during hypoxia [12, 13]. Mehta et al. have shown that under sustained hypoxic conditions (1–4 hours), ROS production was decreased in nonventilation lung [14]. Consistent with Cheng et al. [16], in current study we found that plasma MDA levels were significantly increased and important antioxidant enzyme SOD activity was decreased in patients during one-lung ventilation (OLV), accompanied with increased shunt fraction and decreased PaO2, which indicates that the increased oxidative stress may impair the protective effect of HPV. Yamaguchi et al. found that, in the presence of high level of ROS and reduced endogenous SOD, HPV was considerably suppressed in the isolated rabbit lung but was restored after adding exogenous SOD in the perfusate [32]. Together with above results, our findings that Dex decreased MDA level and maintained SOD activity, concomitant with decreased shunt fraction and increased PaO2, suggested that inhibition of oxidative stress and restore antioxidant defense system may be important mechanisms whereby DEX can augment HPV during one-lung ventilation.

The decrease in shunt fraction in the Dex group occurred in conjunction with increase in nitric oxide concentration in the serum, which suggests that the changes in shunt fraction may be particularly caused by increased nitric oxide, an important endothelium-derived relaxing factor. Mam et al. found that nitroprusside caused less relaxation in the pulmonary arteries in hypoxic than in normoxic rats, suggesting decreased responsiveness of vascular smooth muscle cells (VSMCs) to vasodilators [33]. Hakim et al. found nitric oxide did not affect pulmonary vasoconstriction in hypoxia rat lung [34]. Therefore, we postulated that increased serum nitric oxide mainly affects the arteriovenous in the ventilated lung. It has been reported that inhalation of nitric oxide (iNO) decreases the regional pulmonary vascular resistance of ventilated lung area, decreases intrapulmonary shunting, and improves arterial oxygenation [23, 24], while Minamishima et al. [35] and Lang Jr. et al. [36] found that inhalation of nitric oxide can increase serum nitrite and nitrate (NO), and NO may be the most likely candidate for transducing the iNO stimulus to the organs. Therefore, all above results suggested that serum NO may play an important role in improving arterial oxygenation in ventilated lung. It is reported that Dex can induce vasodilation through activation NO synthase (NOS) [37, 38]. In the current study, Dex significantly increased serum NO concentration, concomitant with decreased shunt fraction and increased PaO2, which indicates that Dex may have induced vasodilation in the ventilated lung by enhancing NO release. In the current study isoflurane did not inhibit NO activation after OLV, while studies found that volatile anesthetics inhibit the NO-mediated relaxation in many vascular beds [39, 40], which may partly explain why volatile anesthetics inhibit HPV.

In conclusion, intravenous infusion of the dexmedetomidine along with isoflurane inhalation during OLV inhibits oxidative stress and increases NO concentration, which may represent a mechanism whereby dexmedetomidine attenuates intrapulmonary shunt and improves arterial oxygenation during one-lung ventilation in patients.

## Figures and Tables

**Table 1:** General characteristics and preoperative data.

	DISO group	NISO group
	(*n* = 25)	(*n* = 24)
Age (yr)	55 ± 12	56 ± 11
Gender (M/F)	17/8	16/8
Weight (kg)	61 ± 12	60 ± 14
Height (cm)	167 ± 5	165 ± 7
ASA physical status (I/II)	3/22	2/22
Preoperative blood gas		
pH	7.42 ± 0.02	7.41 ± 0.03
PaO_2_ (mmHg)	79.7 ± 13.7	79.5 ± 14.1
PaCO_2_ (mmHg)	35.5 ± 3.7	34.9 ± 4.1
Hb (g/L)	119.5 ± 13.8	120.5 ± 12.5
Preoperative pulmonary function		
FEV_1_ (%)	79 ± 17	81 ± 16
FVC (%)	86 ± 14	87 ± 11
FEV_1_/FVC (%)	83 ± 13	80 ± 10

FEV_1_: forced expiratory volume in one second; FVC: forced vital capacity; Hb: hemoglobin. There is no statistic difference between groups.

**Table 2:** Perioperative time-course changes of blood gas variables in DISO group (*n* = 25) and NISO group (*n* = 24) ($\overset{-}{x}\pm s$).

Parameters	Group	TLV-15 min	OLV-10 min	OLV-20 min	OLV-30 min	OLV-40 min
pH	DISO	7.37 ± 0.04	7.38 ± 0.03	7.39 ± 0.04	7.40 ± 0.03	7.39 ± 0.04
	NISO	7.37 ± 0.04	7.38 ± 0.03	7.39 ± 0.04	7.40 ± 0.03	7.39 ± 0.04

Hb (mg/L)	DISO	118.5 ± 10.7	116.9 ± 12.5	117.5 ± 11.6	116.1 ± 14.2	115.3 ± 12.5
	NISO	117.4 ± 12.4	117.0 ± 12.3	116.9 ± 14.3	115.9 ± 12.7	115.1 ± 13.4

Qs/Qt (%)	DISO	11.5 ± 1.8	23.5 ± 2.9^ab^	25.3 ± 2.3^ab^	27.1 ± 2.1^ab^	23.5 ± 2.2^ab^
	NISO	12.0 ± 1.1	28.1 ± 2.5^a^	30.1 ± 2.0^a^	31.9 ± 1.9^a^	27.7 ± 2.0^a^

PaCO_2_ (mmHg)	DISO	34.8 ± 3.2	35.0 ± 3.1	35.1 ± 3.9	35.3 ± 4.1	35.1 ± 3.3
	NISO	35.2 ± 3.1	35.7 ± 4.5	36.0 ± 3.7	35.7 ± 4.0	35.5 ± 4.2

SaO_2_ (%)	DISO	99.7 ± 0.1	99.0 ± 1.2	98.7 ± 0.3	98.4 ± 0.8	99.3 ± 0.5
	NISO	99.7 ± 0.4	98.9 ± 0.7	98.4 ± 1.1	97.5 ± 1.5	99.2 ± 0.6

ScvO_2_ (%)	DISO	84.9 ± 9.1	84.5 ± 8.5	83.5 ± 6.9	82.2 ± 9.2	84.4 ± 5.9
	NISO	84.5 ± 8.5	83.6 ± 7.1	81.7 ± 7.3	80.7 ± 9.4	82.7 ± 5.5

HR (beats/min)	DISO	66.3 ± 9.2^b^	65.5 ± 13.1^b^	67.5 ± 12.1^b^	68.7 ± 11.2^b^	67.6 ± 11.3^b^
	NISO	77.5 ± 10.5	78.2 ± 12.8	78.7 ± 13.3	81.5 ± 14.1	78.4 ± 14.3

MAP (mmHg)	DISO	78.9 ± 17.2	77.2 ± 12.5	76.1 ± 10.5	75.0 ± 16.2	76.6 ± 13.4
	NISO	81.1 ± 15.7	79.1 ± 14.2	79.1 ± 14.7	77.5 ± 17.1	77.9 ± 15.3

PaO_2_ (mmHg)	DISO	457.5 ± 85.2	258.6 ± 68.6^ab^	198.5 ± 68.3^ab^	185.6 ± 73.2^ab^	209.6 ± 85.1^ab^
	NISO	461.5 ± 87.5	223.5 ± 89.7^a^	165.2 ± 75.3^a^	151.3 ± 68.5^a^	171.6 ± 88.9^a^

^a^ *P* < 0.05 versus TLV-15 min; ^b^ *P* < 0.05 versus NISO group.

**Table 3:** Changes in MDA level, SOD activity, and NO concentration in DISO group and NISO group ($\overset{-}{x}\pm s$).

	Group	TLV-15 min	OLV-30 min
MDA (umol/L)	DISO (*n* = 25)	16.9 ± 1.7	17.5 ± 1.2^b^
	NISO (*n* = 24)	18.3 ± 1.7	21.0 ± 1.7^a^

SOD (ug/mL)	DISO (*n* = 25)	1.9 ± 0.2	1.8 ± 0.3^b^
	NISO (*n* = 24)	1.9 ± 0.1	1.6 ± 0.2^a^

NO (ug/mL)	DISO (*n* = 25)	1.9 ± 0.3	2.3 ± 0.3^a;b^
	NISO (*n* = 24)	1.9 ± 0.4	1.8 ± 0.1

^a^ *P* < 0.05 versus TLV-15 min; ^b^ *P* < 0.05 versus NISO group.
